# Nanoencapsulation for Agri-Food Applications and Associated Health and Environmental Concerns

**DOI:** 10.3389/fnut.2021.663229

**Published:** 2021-04-08

**Authors:** Dipendra Kumar Mahato, Awdhesh Kumar Mishra, Pradeep Kumar

**Affiliations:** ^1^Consumer-Analytical-Safety-Sensory Food Research Centre, School of Exercise and Nutrition Sciences, Deakin University, Burwood, VIC, Australia; ^2^Department of Biotechnology, Yeungnam University, Gyeongsan, South Korea; ^3^Applied Microbiology Laboratory, Department of Forestry, North Eastern Regional Institute of Science and Technology, Nirjuli, India

**Keywords:** nanoparticle, human health, food and agriculture, environment concern, toxicity

**Graphical Abstract:**
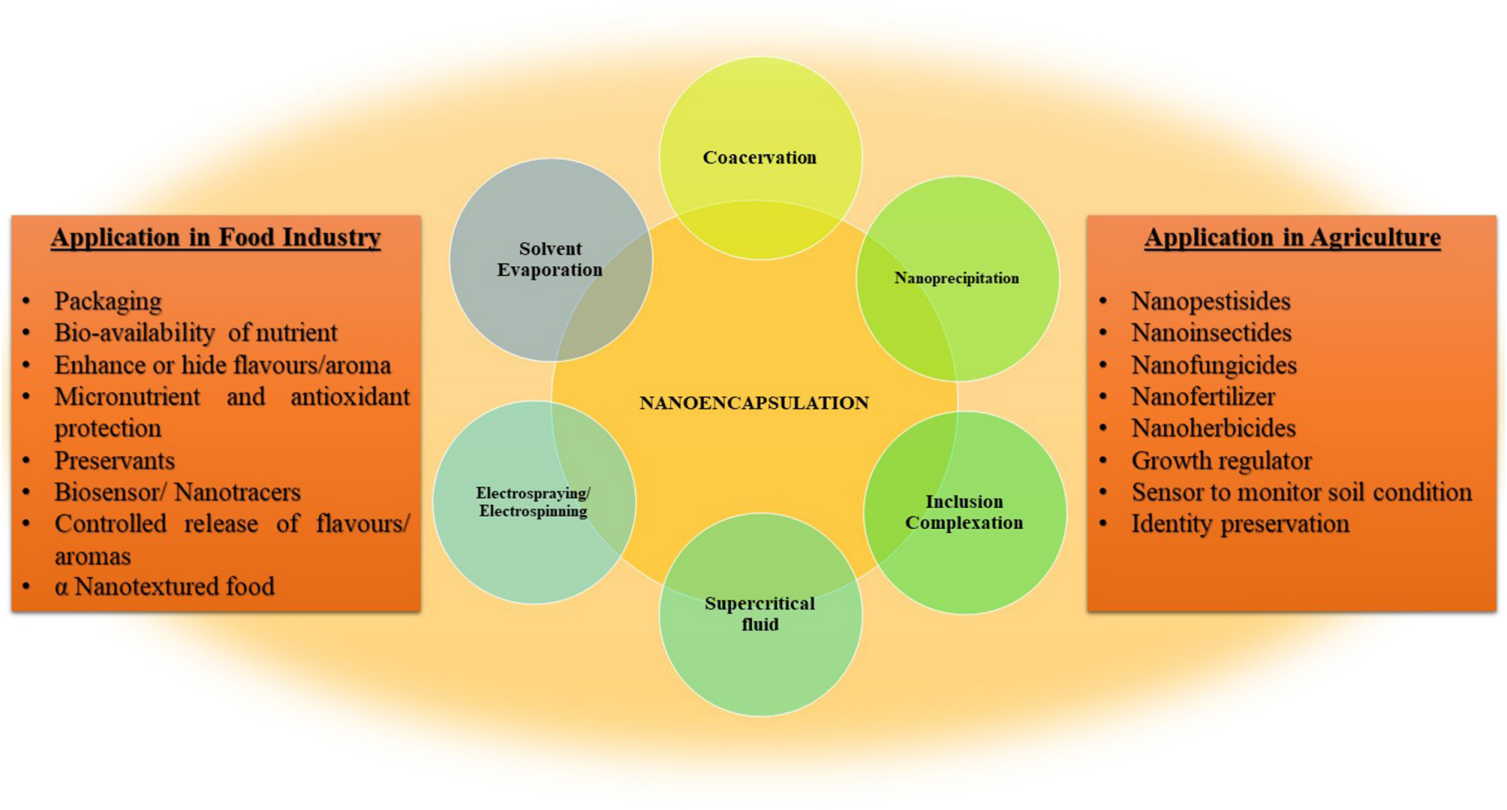
Applications of nanoencapsulation for agri-food industry.

## Introduction

Food safety and security are vital to guarantee a sustainable and reliable energy source for human. There is a recent trend of nano-encapsulating bioactive compounds from both plant and animal sources and their utilization for various food applications (1). Nanoencapsulation has gained special attention because of its unique feature for efficient encapsulation, enhanced stability, and better controlled release of encapsulated materials (2, 3). Nanoencapsulation is also applied for food packaging system with the use of biodegradable polymers reinforced with nanofillers as a sustainable and environmentally friendly option (4). However, the incorporation of compounds into the food packaging system at nanoscale (particle size between 1 and 100 nm) has raised concerns on their migration and release into food matrices and the health effects lying with the consumption of such foodstuffs. Hence, it becomes paramount to study the migration behavior into food matrices and associated toxicity after entering the human body as well as the biodegradability/toxicity and its role in the environment.

Several concerns on the use of nanoparticles (NPs), their release kinetics, absorption behavior in the body, degradation kinetics and their long-term effects are uncertain and unexplored, therefore, in-depth research is required on these aspects to understand the broader figure of the story. Hence, we strongly recommend exploring these aspects to reveal and disseminate the underlying safety concerns associated with the use of nano-encapsulated particles and to avoid any unfortunate and unprecedented outcomes in the future.

## Absorption Behavior of Nanoparticles in the Body

The particles within nanometer range may behave differently within the human body with different biological fate i.e., the levels of absorption, distribution, metabolism, excretion and potential. The biological fate of NPs is dependent on their physicochemical properties (e.g., composition, dimensions, interfacial properties structure and physical state) as well as the changes they undergo while passing the gastrointestinal tract (GIT) (5). For example, the biological fate of lipid NP varies depending on whether it is directly absorbed or normally digested by the human body (6, 7). The smaller indigestible NPs accumulate in organs at a faster rate compared to a larger size. Besides this, metallic (Ag and Au) and inorganic (TiO_2_ and SiO_2_) non-digestible nanoparticles are reported to cross the layer of epithelial cells through various routes such as paracellular, transcellular, or persorption (8). Similarly, mineralo-organic NPs formed from calcium, carbonate and phosphate can lead to ectopic calcification and kidney stones. Further the mineral particles may be involved in the immune tolerance against the gut microbiota and food antigens (9). The NP may be either digested, accumulated or transferred into the systemic circulation via the blood or lymph systems after being absorbed into an epithelium cell (10). NPs may translocate through the human body followed by metabolization, excretion, or accumulation within certain tissues after exiting epithelial cells (6). However, an in-depth investigation is desired to reveal the fate of direct adsorption of both indigestible and digestible lipid NPs in humans.

Fu et al. (11) observed the toxic effects of Ag NPs after being absorbed through the intestine to the liver of the mice when administered orally. The Ag NPs were also reported in spleen and liver when administered intravenously. However, with a lower absorption rate and the NPs were finally excreted through urine and feces. Choi et al. (12) demonstrated that non-cationic surface charged NPs (<34 nm) could efficiently translocate from the lungs to mediastinal lymph nodes. NPs (<6 nm) were rather translocated rapidly from lungs via lymph nodes to the bloodstream and finally cleared by the kidneys. Further, Gerloff et al. (13) reported the cytotoxic with DNA damaging effects of TiO_2_, SiO_2_, ZnO and MgO NPs along with carbon black on human intestinal Caco-2 cells. Alterations in the microbiome in GIT leads to various gut disorders like inflammatory bowel diseases and metabolic syndrome (14). It is speculated that TiO_2_ and Ag NPs may alter the gut microbiota (15). This is attributable to the antimicrobial property of NPs and the production of reactive oxygen species (ROS) (15, 16). However, further in-depth research is required to reveal the absorption behavior and biological fate of various NPs.

## Release Kinetics of Nanoparticles

The release of bioactive compounds is referred to their translocation from one site to another over a time period. Several factors influence the release of NPs, namely (i) thermodynamic factors; (ii) kinetic factors; (iii) chemical structure, particle size and weight of nano-encapsulates; (iv) physicochemical properties like volatility and hydrophobicity; (v) concentration; and (vi) oral processing behavior (17). Release of the bioactive components at the target site known as “Targetability” is another important aspect of release kinetics. This can be achieved through the utilization of liposomes, nanoliposomes or any other nanocarrier systems. The target mechanism can be either active or passive. For example, incorporating antibodies in the lipid carriers is a form of active mechanism while targeting through the particle size of the vesicles is a passive form (18, 19).

Several *in vivo* studies using various NPs demonstrate hazard identification, however, caution should be taken while extrapolating their mechanistic results for hazard characterization and subsequent risk on human health (20). Different NPs have been reported to trigger the release of ROS and subsequently leading to oxidative stress and inflammation via their interaction with the reticuloendothelial system (20). NPs do not bind to the cell membrane but have direct access to the intracellular proteins, organelles and DNA, thereby leading to potential toxicity (21). Having said that, the plausible interactions of NPs with cell components are not fully understood and need further in-depth research. Few studies suggest that NPs may pass the blood-brain barrier but still not clear whether it is a generic effect or shown only by specific subgroup (22). Further, the transfer of NPs across the placenta or via breast milk has the potential for embryotoxicity (23). The data on distribution behavior of NPs in the reproductive cells are insufficient to draw any conclusion. Therefore, investigation should be focused on repro-toxicity of NPs and their passage through the placenta (6).

In addition, after the release of NPs into the environment, surface water forms on the NP's surface creating the entry points and dispersion into soil and soil biota. At this stage, NPs undergo various transformations such as physical, chemical and biological transformations (24). NPs can be bio/geo-transformed in the soil leading to their toxicity, generation of oxidative stress, and absorption by plants that ultimately pose alarming concerns for human health via entering into the food chain (25). NPs get absorbed through roots and then translocate and accumulate to aerial parts via biotransformation and bioaccumulation (26). These scenarios highlight the need for in-depth kinetic studies of NPs and their potential health and environmental concerns.

## Toxicity of Nanoparticles

Among the various compounds, food-grade TiO_2_ is widely used for food applications and hence the safety aspects of its NPs should be evaluated (27). Studies suggest TiO_2_ NPs to be more toxic compared to larger particles of TiO_2_ (28). Oral ingestion of TiO_2_ (>100 nm) has a lower toxicity than TiO_2_ (<100 nm) (29). A study revealed an elevated level of elemental Ti in human blood for 6 h after intake of food-grade TiO_2_ (30) suggesting easy absorption of NPs in the GIT (31). Besides this, TiO_2_ NPs may lead to reproductive issues upon acute oral exposure. For example, Philbrook et al. (32) observed fatality in pregnant mice treated with 100 and 1,000 mgkg^−1^ TiO_2_ NPs. In addition, the effects on cardiac and inflammatory responses (33); blood and bone marrow system (34) were noticed in mice exposed to TiO_2_ NPs.

Another aspect of toxicity lies with how NPs interact with various food components. NPs can interact with food components (e.g., phospholipids, sugars, nucleic acids) and influence the absorption and release kinetics (35). Proteins along with other biomolecules bind and trap NPs which affects their digestion process. Similarly, carbohydrates, fatty acids and proteins play a significant role in the uptake of Ag NPs into Caco-2 cells where food components resulted in enhanced uptake of NPs by 60% (36). In addition, water activity of food impacts the release kinetics of NPs (37). pH and composition of the food also affect the stability, dissolution, and toxicity of NPs (38). Solubility is another crucial factor for the toxicity of NPs. TiO_2_ and SiO_2_ NPs are insoluble while Ag and ZnO NPs are partially/completely soluble in GIT fluids (37). Therefore, uptake and toxicity of soluble NPs (e.g., ZnO NPs) are enhanced (39).

Further, the cytotoxic effects of Ag NPs via altered membrane permeability and integrity has been observed in mammalian cells (40). NPs entering the mammalian cells via endocytosis are translocated to lysosomes, while NPs passing the plasma membrane via diffusion enter the cytoplasm and are less toxic compared to earlier (41). NPs attach to the membrane proteins, damage mitochondria and DNA, produce ROS, alter enzyme activity, integrity and functions of cells (40). The toxicity is a result of Ag NPs interaction with proteins and creation of protein corona with altered functions (42). Even the changes and mutation in DNA may occur (43). However, toxicity varies with the type of NPs and therefore, proper hazard analysis of all types of NPs for food applications are essential (44).

The wide applications of nano-fertilizers and nano-pesticides into the cultivated soils for agri-food production have concerns for their long-term effects which needs to be evaluated (45). For example, it is estimated that 95% of Cu used will eventually end accumulating with a concentration of 500 μgkg^−1^ (46) and ZnO up to 16 μgkg^−1^ (47) in the soil and aquatic sediments. Studies have shown NPs causing damage to the lung in rats by the consumption of TiO_2_ NPs (20 nm) and Fe NPs (48). Further, TiO_2_ NPs potentially damage the brain in dogs and fish (49). Both Ag and TiO_2_ NPs have demonstrated cytotoxic and genotoxic effects due to ROS generation leading to cell proliferation and DNA damage in mice and human cells (50). Therefore, NPs could be dangerous for both human beings and the environment. Hence the application of nanoencapsulation and associated NPs for agri-food application should be tackled with great care and responsibility. The unauthorized and haphazard use of NPs can contaminate both soil and plant systems and ultimately intoxicate the agricultural ecosystem (51).

## Discussion

Nanoparticles are widely used in agriculture and food sector for enhancing the productivity and quality of foods (52). Despite the various positive applications of nano-encapsulated bioactive compounds widely reported by several studies for agri-food applications (Table 1), the associated potential risks for human health should not be underestimated. The mechanisms of release kinetics of nanoparticles from various formulations and production processes need to be characterized and fully elucidated. In addition, the knowledge gap concerning the biological fate, distribution and accumulation of NPs in humans raises concerns for their use and potential toxic effects (6).

**Table 1:** Nanoencapsulation of bioactive compounds for agri-food applications.

**Bioactive compound**	**Nanoencapsulation technique**	**Wall material**	**Application**	**References**
Vitamin B_2_, Vitamin C	Ionic gelation	Alginate/chitosan	Controlled release of vitamins for usage in food industry	(53, 54)
*Satureja hortensis* L. Essential Oil (EO)	Ionic gelation	Sodium trypolyphosphate-Chitosan	As antimicrobial and antioxidant agents with enhanced stability against adverse environmental conditions	(55)
Curcumin	Coacervation	Chitosan	Enhanced antioxidant activity with novel delivery system	(56)
Glucose oxidase	Electrospinning	Polyvinyl-alcohol/chitosan/tea-extract	Novel food packaging system for food preservation	(57)
Eugenol	Emulsion-ionic gelation	Trypolyphosphate-Chitosan	For improved thermal stability and antioxidant activity	(58)
Oil: Medium chain triglyceride	High-pressure homogenized emulsions and layer-by-layer shell assembly	Modified starch- chitosan-lambda carrageenan	Development of nanocapsules for food industries	(59)
Lysozyme	Electrospinning- layer by layer assembly	Chitosan	Preservation of pork against *Escherichia coli* and *Staphylococcus aureus*	(60)
*Lippia sidoides* EO	Spray drying	Angico gum/chitosan	For enhanced release property	(61)
α-tocopherol	Freeze drying	Zein-chitosan	Enhanced stability and protection against environmental conditions	(62)
Lime EO	Nanoprecipitation	Chitosan	Antibacterial properties against food-borne pathogens	(63)

The applications of NPs for food packaging can cause toxic effects upon migration of the NPs from packaging system to the foodstuffs (64). Since nanotoxicology and nanoecotoxicology are novel scientific fields, therefore risk assessments are paramount (65). In addition, the proper rules and regulations need to be set up to check the haphazard and extensive use of NPs without investigating the possible long-term effects on human and animal health. The lack of information on risk assessment and proper regulation highlights the need for further in-depth research (66).

Therefore, with these unanswered questions and valid reasons for health and environmental concerns associated with the use of NPs for agri-food application, we strongly advocate for in-depth and unbiased additional research in the field to ensure safe and sustainable agri-food products. This will further guarantee the safety and security of food and nutrition for human and animal health besides a sustainable environment.

## Conclusion

As concluding remarks, there is an urgency to increase and spread the knowledge and perception on NPs, their beneficial applications as well as associated risk for agri-food applications and how to tackle them to guarantee the safe, healthy and sustainable agri-food and environment for future generations.

## Author Contributions

DKM wrote the original draft. AKM help in editing during article. PK conceptualized the manuscript and did the final editing of all sections.

## Conflict of Interest

The authors declare that the research was conducted in the absence of any commercial or financial relationships that could be construed as a potential conflict of interest.
